# Testing a method of sampling for entomological determination of transmission of *Wuchereria bancrofti* to inform lymphatic filariasis treatment strategy in urban settings

**DOI:** 10.1186/s13071-020-3905-x

**Published:** 2020-01-23

**Authors:** Rogers Nditanchou, Ruth Dixon, Dung Pam, Sunday Isiyaku, Christian Nwosu, Safiya Sanda, Elena Schmidt, Benjamin Koudou, David Molyneux

**Affiliations:** 1 Policy & Programme Strategy, Sightsavers, Cameroon Country Office, BP 4484, Bastos, Yaoundé, Cameroon; 2 Policy & Programme Strategy, Sightsavers, 35 Perrymount Road, Haywards Heath, West Sussex, RH16 3BW UK; 3 Department of Zoology, University of Jos, Bauchi Road, 2084, Jos, Plateau State Nigeria; 4 Sightsavers, Nigeria Country Office, 1 Golf Course Road, Kaduna, Kaduna State Nigeria; 5 Liverpool School of Tropical Medicine, Pembroke Pl, Liverpool, L3 5QA UK

**Keywords:** Lymphatic filariasis, Urban areas, Mosquitoes, Transmission, *Wuchereria bancrofti*

## Abstract

**Background:**

There is on-going debate about scale-up of lymphatic filariasis treatment to include urban areas. Determining *Wuchereria bancrofti* transmission is more complex in these settings and entomological methodologies suggested as a solution as yet have no clear guidance.

**Methods:**

The study was conducted in six communities in Minna and Kaduna cities in Nigeria selected based on pre-disposing risk factors for mosquitoes and Transmission Assessment Survey (TAS) results in 2016 indicating need for treatment (> 1% prevalence). In each community, 4 gravid traps (GT), 15 exit traps (ET) and 21 pyrethrum spray catches (PSC) were used for 5 months targeting a sample size of 10,000 mosquitoes inclusive of at least 1500 *Anopheles*. Community researchers were selected and trained to facilitate community acceptability and carry out collection. We have evaluated the mosquito sampling and trapping methodology in terms of success at reaching targeted sample size, cost effectiveness, and applicability.

**Results:**

Community researchers were influential in enabling high acceptability of the methods of collection and were able to conduct collections independently. Overall, 12.1% of trapping events (one trapping event corresponds to one visit to one trap to collect mosquitoes) were affected by householder actions, weather conditions or trap malfunction leading to lower than optimal catches. Exit traps were the most cost-effective way to catch *Anopheles* (6.4 USD per trapping event and 12.8 USD per *Anopheles* caught). Sample size of 10,000 mosquitoes overall in each city was met though *Anopheles* catch was insufficient in one city. However, sample size was met only in one implementation unit out of the four.

**Conclusions:**

Methods need adapting to maximise *Anopheles* catch: we propose planning 250 gravid trap and 3724 exit trap trapping events in similar settings in West African urban areas where *Culex* is dominant, not using pyrethrum spray catches, and weighting trapping events later in the rainy season. Planning should increase involvement of community researchers, incorporate null catches and participants’ actions to predict catches. Importantly, evaluation units should be analogous with implementation units, the units at which treatment decisions will be made in the urban context.

## Background

Lymphatic filariasis (LF) is a neglected tropical disease (NTD) caused by *Wuchereria bancrofti* in Africa. In West Africa, the dominant vectors are *Anopheles* species [1–3]. LF causes substantial morbidity, disability and stigma and a Global Programme to eliminate the disease as a public health problem commenced in the year 2000. Mass drug administration (MDA) when circulating filarial antigen (CFA) prevalence is > 1% is one element of this programme using albendazole and ivermectin in countries of Africa where onchocerciasis is also endemic. Currently, urban, rural or peri-urban areas inside implementation units (IUs) are treated based on the IU Transmission Assessment Survey (TAS) result. MDA in urban settings presents significant challenges and costs, and often coverage is inadequate [3–7].

While presence of LF in urban areas has been demonstrated, transmission of *W. bancrofti* itself has been a subject of debate [5–7]. Where mapping results have led to treatment in urban areas, elimination was achieved with three or fewer yearly rounds of treatment or without recourse to MDA [7] implying the TAS results may not reflect on-going local transmission [6, 7]. In addition, the findings that West African *Culex*, the most abundant urban mosquitoes appears to be less susceptible to *W. bancrofti*, have led to the hypothesis that urban cases of LF could be due to transient inward migration from rural areas rather than be the result of local transmission [4, 5, 7]. Therefore, the existence of significant transmission of *W. bancrofti* in urban areas needs to be ascertained beyond the currently recommended TAS.

Alongside adapting a confirmatory mapping protocol based on serological surveys [10, 11], xenomonitoring (detecting L3, the infective larval stage of *W. bancrofti*), is being considered as an option for assessing transmission in urban settings [7, 9] as it has the advantage of providing specific transmission information on time and place [8–11]. At present, there is no recommended protocol adapted to the scale of mosquito collection required for this purpose [9, 10]. As a step to informing and developing such a protocol, we present here the methods and results of mosquito collections intended for the estimation of L3 infectivity rates of *W. bancrofti* in mosquitoes as laboratory processing progresses.

## Methods

### Data collection

The study was conducted in six communities in Minna and Kaduna City in Nigeria. Kaduna city is the capital of Kaduna State and lies between latitudes 10°25′15″N and 10°36′08″N and longitudes 7°23′31″E and 7°29′33″E. Based on the 2006 national census [12], the Kaduna metropolis, comprising of Kaduna North, Kaduna South, parts of Chikun and Igabi Local Government Areas (LGAs), has a population of about 1,139,578 and covers an area of around 131 km^2^. The indigenes of the state include the Hausa, Fulani, Gwari, Jaba, Agorok, Atyap and Bajju. In addition, it is home to minorities from all parts of the country. The occupation of the people in the city are mainly commerce and petty trading. Those in the outskirts of the city engage in farming. Minna is the capital of Niger State and has land area of about 1664 km^2^ with a population of 348,430 [12] and lies between latitudes 9°37′N and 9°79′N and longitudes 6°16′E and 6°65′E. Minna metropolis cuts across two LGAs (Bosso and Chanchaga). It is inhabited by two major ethnic groups: the Nupe and the Gwaris who are mostly Christians and Muslims. The major activity of the people of the state is subsistence farming.

The data collection team in each community included two entomologists from the University of Jos, one laboratory technician from the Federal Ministry of Health and two community researchers. These community researchers were selected through the community leaders based on their sociability and a literacy level that allows for recording data. Using a step-by-step purposely developed implementation protocol, they were trained to guide locations of suitable sites in their communities, facilitate engagement with the communities and households as well as assist in collecting mosquitoes and recording data.

Communities were selected based on TAS results indicating prevalence of *W. bancrofti* > 1%, belonging to an urban slum and safe for the research team (Table 1). Due to the scaling-up of the National NTD Programme in the two states, all areas with LGA level LF antigen prevalence meeting the > 1% threshold had started treatment. However, those with minimum years of treatment of LF were selected (two or fewer years) for the study. Following selection of communities, collection sites, usually the households, were selected based on their proximity to pre-disposing risk factors for mosquitoes (swamps, rice fields, poor drainage, congestion, poor housing, streams/rivers, and wastewater).

**Table 1:** Characteristics of selected communities

State	LGA	No. of communities selected	Baseline (2016) ICT LF prevalence (%)	No. of years of MDA for LF
Kaduna	Kaduna South	2	14	2
Kaduna	Kaduna North	1	2	2
Niger	Chanchaga	1	4	2
Niger	Bosso	2	2	2

Among the households or areas near breeding sites, 41 were systematically selected in each community for mosquito trapping events. Four gravid traps (GT) utilising 3-day-old dry grass fermented water as attractant were placed outside the selected household/compounds at or around sunset and emptied at sunrise the following day for 7 days a month (reduced days towards end of study due to overcollection of *Culex* mosquitoes). Batteries were changed every 2–3 days, and the condition of the trap (if the fan was still running, any flooding of the trap) was recorded at each collection. Fifteen exit traps (ET) were attached to windows of sleeping rooms for 10 days a month and emptied daily by aspiration prior to 8:00 h. At the time of emptying the trap, a short interview was conducted with each householder to confirm the number of those who slept in the room as well as the use of any repellent or insecticide-based products.

Twenty-one pyrethrum spray catches (PSC) household sites were selected. Three PSCs were conducted before 8:00 h in the morning each day for seven days. Selected PSC households were requested to ensure windows and doors remained closed until the collecting team arrived. White sheets were placed over floors and furniture and the room sprayed with insecticide, allowing 10–15 min for the insecticide to take effect before a torch was used to locate mosquitoes which were collected with tweezers. At PSC sites, the team recorded if windows and doors were closed on arrival, and confirmed the number of people who slept in the room, the use of other insecticides, repellents, window screens, mosquito nets and fans. Table 2 shows the details of these methodologies used.

**Table 2:** Mosquito collection methodologies used

Method	No. of sites per community	Description	Target trapping events per community
Exit traps	15 households	Mosquito collection for 10 days each month for 5 months	750
Pyrethrum spray catch	21 households	Three households sprayed each day for 7 days; each household had only one spray catch per month for 5 months	105
Gravid traps	4 outdoor mosquito collection points	7 days collection per month for 5 months, near households or open breeding site	140

Mosquitoes were collected over 5 months, from May to September 2018, coinciding with the high transmission period for malaria. Detailed collection schedules were provided to each household, and prior to collection commencing each month, community researchers visited households to re-affirm their consent and remind them of the upcoming collection. An estimated 10,000 mosquitoes with at least 1500 *Anopheles* were targeted to be collected per city in order to calculate a maximum estimate of the prevalence of L3 of *W. bancrofti* in mosquitoes as recommended by the World Health Organisation (WHO) [9].

### Data management

Data were collected on smartphones using an application running on the Commcare platform (https://www.dimagi.com/commcare/). After each collection, all mosquitoes were counted and sorted by sex, species and abdominal condition. In addition, GPS coordinates, distance from suspected breeding site, travel and treatment history were recorded. The direct costs of implementation were tracked in order to calculate cost per mosquito collected for the each of the three trap types. Data collection was heavily supervised, and collections were tracked daily with regular verification of data uploaded on the Commcare platform to ensure completeness. All entomologists were trained and supervised by two senior entomologists to ensure good data quality and correct identification of mosquitoes. At the end of collection, data were downloaded into Excel before importing into Stata software Version 15.1 (StataCorp LLC, College Station, Texas 77845 USA; http://www.stata.com) for further cleaning and analysis. The effectiveness of traps (reaching target 10,000 per city), feasibility, cost-effectiveness (direct cost per mosquito caught) and applicability (number of mosquitoes caught per implementation unit) of the methods are the focus of this paper.

## Results

In total 36,880 female mosquitoes were collected, including 33,978 (92%) *Culex*, 2818 (7.6%) *Anopheles*, 47 (0.1%) *Mansonia* and 37 (0.1%) *Aedes*. The most productive trap type was GT with a mean catch per trapping event of 64.9, followed by PSC (3.5) and ET (2.1). For the two most prevalent mosquito species, GT had the highest *Culex* mean catch of 64.8 and PSC had the highest mean *Anopheles* of 1 per trapping event. The majority of *Anopheles* (77%) were caught in ETs due to the higher frequency of these trapping events compared to PSC. Seventy-seven percent (28,499) (59% of *Anopheles*, 79% of *Culex*) were either gravid (65%), semi-gravid (5%) or fed (8%) implying they have had contact (a blood meal) with humans (Table 3) and are epidemiologically important as they could contain *W. bancrofti* at various larval stages, if people are infected and transmission is on-going. Twenty-three percent were unfed and may or may not have had a blood meal.

**Table 3:** Mosquito catches by trap type, species and abdominal status

Species	Characteristic	PSC *n* (%)		Exit trap *n* (%)		Gravid trap *n* (%)		All traps *n* (%)	
	No. of trapping events	614		4420		393		5427	
	No. of null trapping events	251 (41)		2273 (51)		22 (6)		2546 (47)	
	No. of mosquitoes	2126 (6)		9235 (25)		25,519 (69)		36,880 (100)	
	Unfed	237(11)		4908 (53)		3236 (13)		8381 (23)	
	Fed	1226 (58)		1366 (15)		246 (1)		2838 (8)	
	Gravid	321 (15)		2103 (23)		21,433 (84)		23,857 (65)	
	Semi-gravid	342 (16)		858 (9)		604 (2)		1804 (5)	
	Mean catch	3.5		2.1		64.9		6.8	
	*Anopheles*	1.0		0.5		0.1		0.52	
	*Culex*	2.4		1.6		64.8		6.26	
*Anopheles* (7.8%)	Total	626 (22)		2156 (77)		36 (1)		2818 (100)	
	Unfed	91	15	1044	48	21	58	1156	41
	Fed	363	58	538	25	4	11	905	32
	Gravid	95	15	363	17	9	25	467	17
	Semi-gravid	77	12	211	10	2	6	290	10
*Culex* (92.0%)	Total	1499 (4)		7019 (21)		25,460 (75)		33,978 (100)	
	Unfed	146	10	3829	55	3205	13	7180	21
	Fed	863	58	814	12	236	1	1913	6
	Gravid	225	15	1729	25	21,417	84	23,371	69
	Semi-gravid	265	18	647	9	602	2	1514	4
*Aedes* (0.1%)	Total	0 (0)		23 (62)		14 (38)		37 (100)	
	Unfed	0	0	16	70	9	64	25	68
	Fed	0	0	4	17	5	36	9	24
	Gravid	0	0	3	13	0	0	3	8
	Semi-gravid	0	0	0	0	0	0	0	0
*Mansonia* (0.1%)	Total	1 (2)		37 (79)		9 (19)		47 (100)	
	Unfed	0	0	19	51	1	11	20	43
	Fed	0	0	10	27	1	11	11	23
	Gravid	1	100	8	22	7	78	16	34
	Semi-gravid	0	0	0	0	0	0	0	0

Ten trapping sites, all of which were PSC sites, caught no female mosquitoes throughout the collection period. The remaining (successful) sites were classified, depending on the percentage of trapping events that caught mosquitoes into low (< 25%), medium (25–75%) and high (> 75%) success traps. Forty percent (39.7%) of PSC, 13.3% of ET and 100% of GT sites were highly successful while 40.5% of PSC and 66.67% of ET were of medium success. Trapping events in sites that were situated less than 6 m from an open breeding site were 1.8 (*χ*^2^ = 92.24, *df* = 1, *P* < 0.001) times more likely to collect mosquitoes for GT and 3.0 times for ET than those over six meters (*χ*^2^ = 6.15, *df* = 1, *P* = 0.01).

**Fig. 1:**
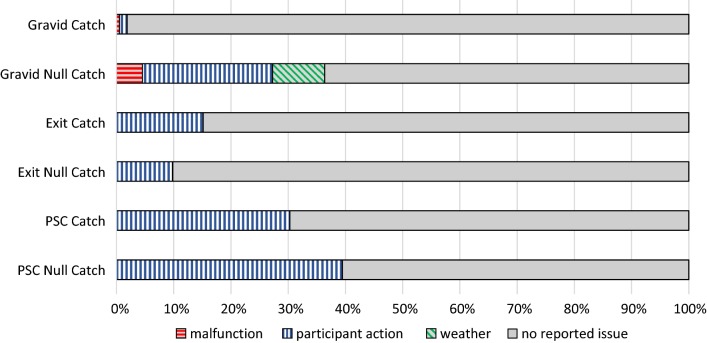
Graph showing breakdown of sub-optimal collection conditions

**Fig. 2:**
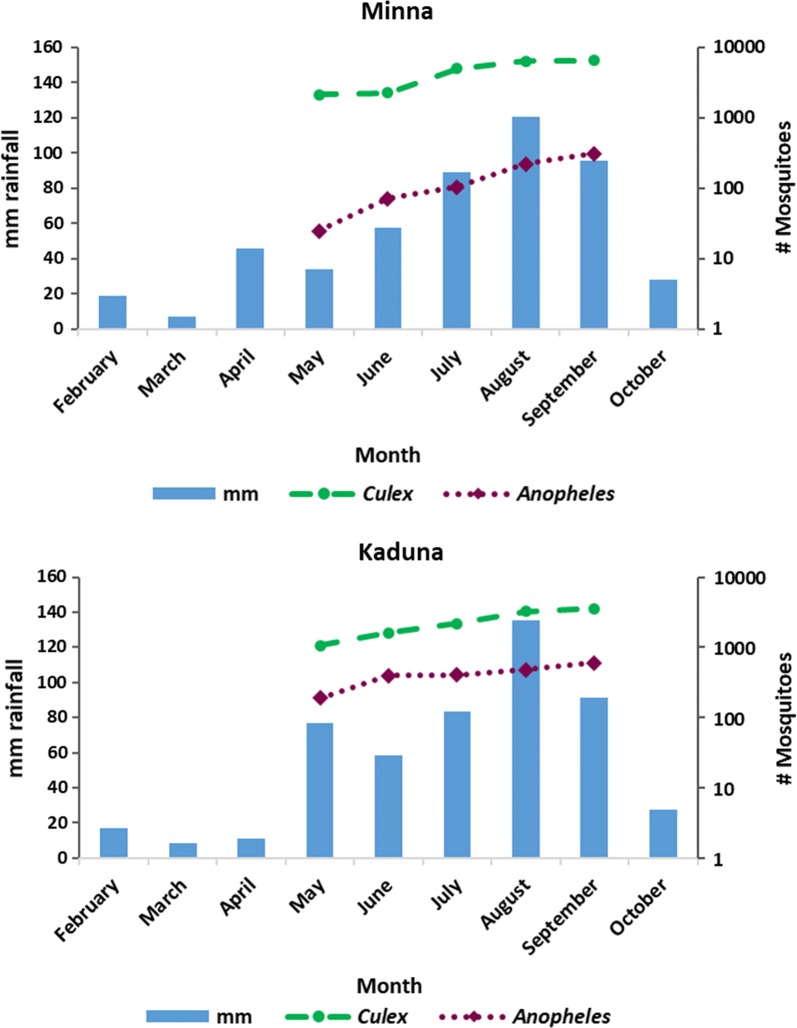
Graphs showing C*ulex* and A*nopheles* mosquitoes collected per month with rainfall data [13] over the period of data collection for Minna and Kaduna

Out of 5427 trapping events, 47% (2546) caught no mosquito. Fifty-seven percent (1448/2546) of these occasions were from sites located at more than 6 m from a breeding site while either trap malfunction, weather conditions or participant actions had impacted 11% (283) of them. Overall, trap malfunction, weather conditions and participant actions affected 12.11% (657/5427) of the trapping events resulting in a potentially sub-optimal collection. Participant actions were important for PSC and ET, while weather and trap malfunction were most important for gravid traps (Fig. 1 and Table 4). For the two main genera of mosquitoes, collections varied over time, with *Anopheles* increase more pronounced than *Culex* from May to September (Fig. 2) as the rainfall increases [13]. Six percent (326) of the collections were undertaken by community researchers corresponding to 12% of PSC, 2.7% of ET and 39% of GT.

**Table 4:** Description of sub-optimal collection conditions

Trap types	Malfunction	Participant action	Weather
Gravid	Issues with the batteries where they ran out of power or were old, meaning the fan was running slowly or intermittently or not running by the time the team arrived	Traps were damaged by animals (goats, sheep) present on people’s properties, usually resulting to trap malfunction due to fan stopping because of disconnection or collapse of the whole trap structure	Strong winds and rains caused water to fill the basins. These caused fans to stop running and prevented entry of more mosquitoes into the trap as well as damaged attractant in the traps
	Disconnected battery		
	Fan running in the opposite direction pushing mosquitoes downward. This indicates that the traps were assembled incorrectly		
Exit	Spiders inside the trap could have eaten mosquitoes	Nobody slept in the room (usually due to heat) meaning mosquitoes would have been less likely to enter the room	Strong winds detached some traps from the window
		People slept under bednets preventing mosquito bites	
PSC		Nobody slept in the room meaning mosquitoes would have been less likely to enter the room and/or resting there	
		People slept under bednets meaning mosquitoes potentially would fail to bite and/or go to rest	
		Use of coil/insecticides reduced the number of mosquitoes in and around the house	
		Some doors/windows were opened on arrival. As a result, mosquitoes would have been able to escape

The target sample size (10,000 including 1500 *Anopheles*) was reached in Kaduna but not in Minna. At the implementation unit level (LGA), the sample size was met in one out of the 4 LGAs in Kaduna City. In Minna, a further LGA and one community reached the overall mosquitoes sample size but not the required number of *Anopheles* (Table 5).

**Table 5:** Sample size success at different (potential decision making) levels

Level of collection	*Anopheles*	All mosquitoes	*n*
City			
Kaduna	2083	13,944	3
Min4na	735	22,936	3
LGA—Implementation unit (urban slum)			
Kaduna South (Makera)	1578	9963	1
Kaduna North (Kabala Doki)	505	3981	2
Bosso (Maitunbi)	531	17,345	2
Chanchaga (Tudun Wada)	204	5591	1
Community			
Down Qaurters	1273	5982	1
Tundun Muntira	305	3981	1
Kabala Doki	505	3981	1
Ungwan Kadara	341	6512	1
Ungwan Muʼazu	190	10,833	1
Tundun Wada	204	5591	1

*Abbreviation*: n, number of communities sampled

Based on the cost of the trappings equipment, number of trapping events and mean mosquitoes caught, ETs were the most cost-effective method to catch *Anopheles* (6.4 USD per trapping event and 12.7 USD per *Anopheles* caught), while GTs were the most cost-effective for *Culex* (13.3 USD per trapping event and 0.2 USD per *Culex* caught) (Table 6).

**Table 6:** Costs of trapping

Activity	PSC	Exit	Gravid
Traps bought^a^	6	90	24
Total events	614	4420	393
Total trap cost (USD)^b^	120	13,500	4800
Trap cost per trapping event (USD)	0.2	3.1	12.2
Trapping events in 1 day	3	15	5
No. of entomologists per day	1	1	0
No. of technicians per day	1	0	0
No of community researchers per day	1	1	1
Team cost per day (USD)^c^	60.0	49.5	5.5
Team cost per trapping event (USD)	20.0	3.3	1.1
Total cost per trapping event (USD)	20.2	6.4	13.3
Mean catch *Anopheles*	1.0	0.5	0.1
Mean catch *Culex*	2.4	1.6	64.8
Cost per *Anopheles* (USD)	20.2	12.7	133.1
Cost per *Culex* (USD)	8.4	4.0	0.2

^a^For PSC this is the cost of fixed costs (lamp, white sheets, forceps, sprayer, facemasks) and supplies (insecticide)

^b^Does not include shipping costs which was provided in kind

^c^For gravid and exit traps this includes a proportion of one day per collection period for trap set up day

## Discussion

The results of this study have a number of implications that should be taken into account in entomological transmission assessments and their interpretation in urban settings. The mean catches provide a starting point for making better catch predictions in similar environments (West Africa cities, slums environments, *Culex-*dominant areas). Based on the mean catches, transmission assessments should plan to accommodate 250 GT and 3724 ET trapping events in settings where *Culex* is most abundant in order to achieve required sample size. This takes into consideration expected trap malfunction, weather conditions and expected participant behaviour as found in this study. Furthermore, site selection should be biased toward open breeding sites and number of traps gradually increased so that areas favourable to collection of *Anopheles* species can be better identified and traps placed there rather than seeking to move established traps.

From a logistical perspective the collection methods were feasible; 98% of collections were completed prior to 8:30 h and 66% of them before 7:30 h. Importantly for such studies, there was high level of community acceptability of all the trapping methods. The community researchers were valuable in facilitating the collection and were also able to empty especially gravid traps as also reported in Ghana [15]. Some traps which failed to collect mosquitoes could not be removed as once enrolled, community members continued to participate in the activities and resisted dropping out of the study.

The timing of the trapping events should also be adapted to maximise *Anopheles* catches if as predicted from other studies in West Africa, *Culex* is an inefficient vector and its epidemiological role in urban West Africa filariasis epidemiology can be discounted [7]. By the peak of rainy season all exit traps should be in place to maximise *Anopheles* catches and catching periods could be extended to further increase the number of trapping events. In the case that *Anopheles* are being collected in sufficient numbers, the number of collection days can be reduced, and extra traps reserved. Considered alongside the potential inconveniencies, insecticide resistance [14], ethical and health issues related to PSC, future collections should focus on exit traps to collect sufficient *Anopheles* mosquitoes for transmission assessment.

With this method of site selection, it is not clear at what level (cities, LGA or communities) the results from the mosquito analysis will be applicable for treatment decisions. Primarily, this is because the evaluation unit (city) used here does not correspond with implementation units at which treatment decisions are currently made (LGA). Since urban areas can cross multiple implementation units but represent a small part of each, redefinition of feasible implementation units may be required and in this situation evaluation units should correspond to those. Also, given localised transmission patterns and a maximum flight range of 3 km for mosquitoes, the sample can at best apply to 3 km diameter area [11], where transmission may be occurring but difficult to apply to an evaluation unit which is wider than 3 km. This is because sample size used here, recommended by the WHO [9], has not specified the size of the areas (what geographical unit should the 10,000 mosquitoes be collected from or which implementation unit the results will apply to) the results can reflect. In the light of this, it is important to further refine sampling protocols that are programmatically useful, cost-effective and feasible.

Future studies should focus on operational research assessing the evaluation unit to which 10,000 *Culex* and 1500 *Anopheles* mosquito collection target apply with consideration given to implementation units at which a treatment decision can be reasonably applied. Entomological and geospatial analysis will be useful to understand a maximum geographical area (transmission zone) to which the results in terms of identified transmission can be applied and most appropriate sampling structure. Detailed mapping is also advised utilising available data on malaria transmission, mosquito densities, presence of slum areas and breeding sites (rivers, lakes, agricultural areas) and areas of ‘green’ prior to applying any sampling strategy or community selection.

## Conclusions

This study reports the protocols and results of the deployment of three trapping methods used to collect mosquitoes to assess the transmission of *W. bancrofti* in two urban settings in Nigeria where it was necessary to evaluate ongoing transmission. *Culex* species were the most abundant mosquitoes caught and the study also identified that exit traps were the most effective method of trapping the putative vector in urban areas, *Anopheles*. It is important in future to concentrate studies on the use of exit traps to ensure the maximum numbers of *Anopheles* are collected in unit time. We suggest a starting number of trapping events to assist other entomological transmission assessments to reach the current target mosquitoes as cost-effectively as possible. The study also demonstrated the value of engaging the local community in assisting mosquito collections as their role in improving acceptability and gaining access to households in complex settings was also critical. In light of the increased trapping events required in these *Culex-*dominant settings, community researcher involvement could also increase significantly the cost-effectiveness of such assessment.

## Data Availability

The datasets analysed during the present study is available from the corresponding author upon reasonable request.

## Abbreviations

MDA: mass drug administration
LGA: local government area
TAS: transmission assessment survey
CFA: circulating filarial antigen
GT: gravid trap
PSC: pyrethrum spray catches
ET: exit trap
LF: lymphatic filariasis
L3: *Wuchereria bancrofti* larval stage 3
NTD: neglected tropical diseases
GPS: global positioning system
WHO: World Health Organisation
